# Machine Vision-Based Method for Measuring and Controlling the Angle of Conductive Slip Ring Brushes

**DOI:** 10.3390/mi13030447

**Published:** 2022-03-16

**Authors:** Junye Li, Jun Li, Xinpeng Wang, Gongqiang Tian, Jingfeng Fan

**Affiliations:** 1 Ministry of Education Key Laboratory for Cross-Scale Micro and Nano Manufacturing, Changchun University of Science and Technology, Changchun 130022, China; ljy@cust.edu.cn (J.L.); 2019100431@mails.cust.edu.cn (J.L.); 2020100502@mails.cust.edu.cn (X.W.); qjy@mails.cust.edu.cn (G.T.); 2 Chongqing Research Institute, Changchun University of Science and Technology, Chongqing 401135, China

**Keywords:** machine vision, image processing, angle measurement, brush wire rebound, conductive slip ring

## Abstract

The conductive slip ring is used for power or signal transmission between two objects rotating relative to each other. It has become an essential part of modern industrial development. In traditional automated production measurements, the typical method is to use calipers, goniometers, or angle gauges to measure a parameter of the workpiece several times and then average it. These inspection means have low measurement accuracy and slow measurement speed, and measurement data cannot be processed in a timely manner. A machine vision-based method for measuring and controlling the angle of the brushes is proposed for this problem. First, the brush angle forming device was built for the conductive slip ring brush wire, forming the principle and rebound characteristics. Then, machine vision and image processing algorithms were applied to measure the key parts of the conductive slip ring brushes. The data of the forming angle value and rebound angle value were obtained during the forming process of the brush wire angle. Finally, a pre-compensation model for the brush filament rebound was developed and validated based on the curve fitting method. The test results show that the error of the angle measurement is within 0.05°. The average error of the measured rebound angle and the calculated rebound angle of the brush filament pre-compensation model was 0.112°, which verifies the correctness of the pre-compensation model. The forming angle can be controlled more precisely, and the contact performance between the brush wire and the ring body can be improved effectively. This method has the potential to be extended to engineering applications.

## 1. Introduction

Conductive slip rings are key components of various precision rotary tables, centrifuges, and inertial guidance equipment and have been used in sophisticated military applications [1]. In the context of today’s Industry 4.0, there are higher requirements for the signal transmission rate and rotation speed of conductive slip rings. Technical indicators are gradually approaching the direction of zero wear and zero maintenance [2]. Therefore, scholars have conducted in-depth research on the contact form [3,4,5], material wear [6], and performance reliability of conductive slip rings [7]. The position of the brush wire in contact with the slip ring directly affects the amount of pressure in contact with the slip ring. When the contact pressure is too small, the resistance of the contact becomes large, the temperature rise is large, and the loss also increases, which in turn affects the current as well as the signal transmission. If the pressure is too large, it will cause the brush to increase the amount of wear and tear, resulting in a melt welding phenomenon that affects the service life of the conductive slip ring [8]. As the brush wire is mounted on the brush plate, when measuring the brush wire by mechanical contact, it affects the form and surface quality of the brush wire, and the uneven surface on the brush plate will bring some error to the measurement result. Therefore, it is necessary to find an optimal angle measurement method.

Many scholars have conducted a series of studies and analyses on machine vision-based measurement technology because of its non-contact, high stability, high speed, and low-cost characteristics [9]. Some scholars have focused on practicalizing vision detection on the one hand, such as medical detection, fruit and vegetable quality inspection, navigation, and other directions. Sánchez-Jiménez et al. [10] presented research advances in two computer vision techniques for skin wound measurement, using an open-source computer vision library (OpenCV) and SciPy open-source libraries to calculate skin wound dimensions in an objective and reliable manner, allowing the generation of two-dimensional results in real time and the submission of the results as a report to medical experts for interpretation as a complementary test. Blasco et al. [11] presented a quality assessment monitoring system based on machine vision and optical techniques. This system is much faster than any manual nondestructive inspection of the quality of fruits and vegetables. The detection of color, shape, and external defects is based on machine vision for color images and finally provides a better assessment of ripeness and other defects. Eriksen et al. [12] proposed a visual ranging algorithm for underwater navigation based on monocular cameras. He evaluated the performance of the proposed algorithm on underwater video sequences and discussed how to further improve the robustness and computational performance of the algorithm. On the other hand, some scholars have improved the visual measurement calibration detection algorithm to improve the accuracy and stability of detection. Gadelmawla et al. [13] presented a vision system for the automatic inspection of various threads. The system was calibrated for imperial and metric units, and the measurement results were compared with the standard values by measuring standard metric thread plug gauges. The results show that the vision system has good inspection accuracy. Du et al. [14] proposed a fast and effective method for detecting passive components and providing complete measurements and sufficient accuracy in automatically detecting the physical characteristics of passive components. Kewei et al. [15] proposed a calibration-based method for optical planimetry. He used photogrammetry in computer vision to display six different orientations of the inspection graphics on another Liquid Crystal Display (LCD) and calibrated the pinhole camera. He obtained the intrinsic parameters of the pinhole camera by processing the images of the inspection graphics. Hacini et al. [16] proposed a one-dimensional numerical fractional-order Charef differentiator (FCD). It was generalized to 2D by means of multi-directional operators, and the 2D operator was eventually improved by defining x and y multi-directional operators. He proposed 2D-IFCD-based edge extraction and conducted numerical experiments to evaluate the performance of 2D-IFCD-based edge extraction methods, and the obtained results proved its effectiveness. Othman et al. [17] proposed a method to calculate pixel thresholds for foreground and background images from global and local image analysis. According to this method, an image is divided into blocks according to different resolution levels and then an improved sampling method is applied globally and locally. The results show that the method outperforms the Canny method and other adaptive methods.

Some other scholars have also researched the advantages of vision inspection for processing and manufacturing as well as practical applications, optimizing and improving the accuracy of inspection algorithms and measurement methods. Dong et al. [18] has made great progress in the field of structural health monitoring (SHM) by combining machine vision-based sensing technology with digital image processing algorithms. Sun et al. [19] used this technology to effectively identify and classify weld defects of thin-walled metal canisters. A weld defect detection and classification algorithm based on machine vision is proposed. Li et al. [20] proposed a novel defect extraction and classification scheme for mobile phone screens based on machine vision. A clustering algorithm is proposed to avoid the false detection or missed detection of cluster defects. Xie et al. [21] proposed an improved image sequence velocity detection algorithm combined with the Kalman prediction equation to predict the coordinate position of the workpiece in the nonlinear motion state in order to compensate the time delay error of the visual servo system. Finally, an experiment was carried out on a visual tracking robot system with a conveyor belt. The results show that the maximum tracking speed can reach 90 mm/s, and the tracking accuracy error is less than 1 mm. Wang et al. [22] presented a new deep learning-based machine vision inspection method to identify and classify defective products without a loss of accuracy. Dong et al. [23] developed a machine vision-based system for detecting the position of micro-slots on the surface of conductive slip rings in order to accurately detect the position of micro-slots on the surfaces of conductive slip rings.

In the following part of this paper, we first explain in detail the built brush wire angle forming and measuring device and image pre-processing. A machine vision-based brush angle measurement and control method is proposed. Then, we measure the brush wire forming angle and rebound angle value data under the autonomous control of the forming angle value, build a data pre-compensation model, and experimentally verify this model. It can control the forming angle more precisely and improve the contact performance of the brush filament and the ring body effectively. A detailed discussion based on the results and observations is presented. The accuracy and applicability of the machine vision-based brush wire angle measurement method are demonstrated by comparisons with experiments. Lastly the paper summarizes its conclusions, and potential further studies are suggested.

## 2. Principles and Methods

### 2.1. Brush Wire Angle Forming and Measuring Device Construction

**Figure 1:**
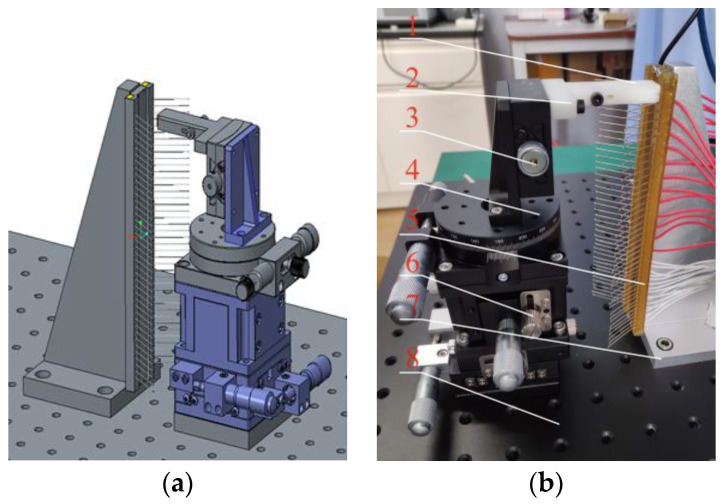
Design of the brush wire forming device. (**a**) Three-dimensional diagram of the brush wire angle forming device; (**b**) Brush wire angle forming device.

A brush wire angle forming device was designed for the phenomenon of rebound and uneven force during angle forming of brush wire. The device consists of a clamping plate (1), a deflection plate (2), an angle table (3), a rotation table (4), a brush plate (5), a displacement table (6), a positioning plate (7), and an optical platform (8). As shown in Figure 1, the angle of the brush wire is shaped by designing the brush wire clamping device using the torque transmitted by the deflecting angle stage and the accuracy of the deflecting angle.

**Figure 2:**
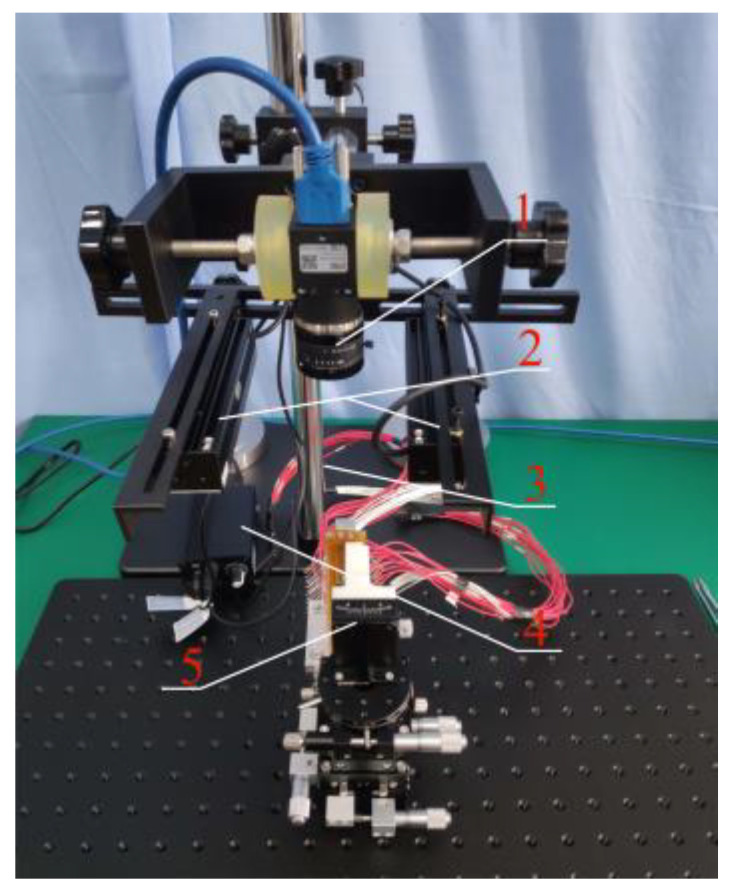
Brush angle measuring device.

The brush wire angle measuring device is shown in Figure 2. The device consists of a camera (1), two light sources (2), a support frame (3), a light source controller (4), and an angle forming device (5). In this paper, we chose the model MER-500-14U3M CMOS black-and-white surface array camera of the Daheng Mercury (Beijing, China) series with a resolution of 2592 × 1944 and a mechanical size of 29 mm × 29 mm × 29 mm, which is compact in size and convenient to install. The pixel size is 2.2 μm × 2.2 μm. The selected focal length of the lens should have been 8 mm, and positive illumination was selected. In order to avoid the generation of positive lighting shadows, we selected symmetrical lighting to offset the generation of shadows. The LED light source was selected, and the light source consisted of a controller and two strip light sources.

### 2.2. Camera Calibration

#### 2.2.1. Camera Aberration Model

The photographed object in the imaging process will produce a certain amount of aberration, resulting in deviations in visual measurement results. The aberration of the camera is mainly divided into radial aberration and tangential aberration [24].

(1) Radial distortion

The radial distortion in the optical center of the camera is 0. As it moves outward, the radial distortion increases symmetrically in either direction. Therefore, radial distortion is more easily observed at the edge of the image. The straight lines near the image edge are bent due to radial distortion, a phenomenon of expansion known as the barrel or fisheye effect. Radial distortion can be expressed in the form of a Taylor series, with the first two terms of the series typically used to approximate the radial distortion phenomenon. The pixel coordinates of the image (*x*, *y*) will be distorted according to the following factors:

(1) $$x_{d}=x\left(1+k_{1}r^{2}+k_{2}r^{4}+k_{3}r^{6}\right)$$

(2) $$y_{d}=y\left(1+k_{1}r^{2}+k_{2}r^{4}+k_{3}r^{6}\right)$$

where $r=\sqrt{{\left(x-x_{p}\right)}^{2}+{\left(y-y_{p}\right)}^{2}},\;k_{1}$, $k_{2}$, $k_{3}$ are the radial distortion coefficients of the camera lens. x and y are the coordinates of the pixel. $x_{p}$ and $y_{p}$ are the coordinates of the center of the aberration. Generally, the $k_{1}$ accuracy can satisfy the aberration correction application for a narrow field of view. In this paper, the third-order accuracy of $k_{1}$, $k_{2}$, and $k_{3}$ was chosen to ensure the accuracy of the acquired graphics.

(2) Tangential aberration

During the manufacturing process of a camera, the lens may not be perfectly parallel to the imaging plane. As a result, this manufacturing defect usually causes tangential lens aberrations, where the coordinates of the pixels are distorted.

(3) $$x_{d}=x+\left(2p_{1}y+p_{2}\left(r^{2}+2x^{2}\right)\right)$$

(4) $$y_{d}=y+\left(p_{1}\left(r^{2}+2y^{2}\right)y+2p_{2}x\right))$$

Where $p_{1}$ and $p_{2}$ are the tangential aberration parameters of the lens.

#### 2.2.2. Calibration Methods

The general camera calibration is divided into the linear calibration method, nonlinear calibration method, two-step method, and tensor calibration method [25]. The Zhang calibration method combines the advantages of linear calibration and nonlinear calibration, and the method is simple to operate and has high accuracy, which is suitable for most occasions. In this paper, Zhang’s calibration method was be used for camera calibration. The aberrations of the acquired feature images are corrected by the OpenCV tessellation calibration function.

**Figure 3:**
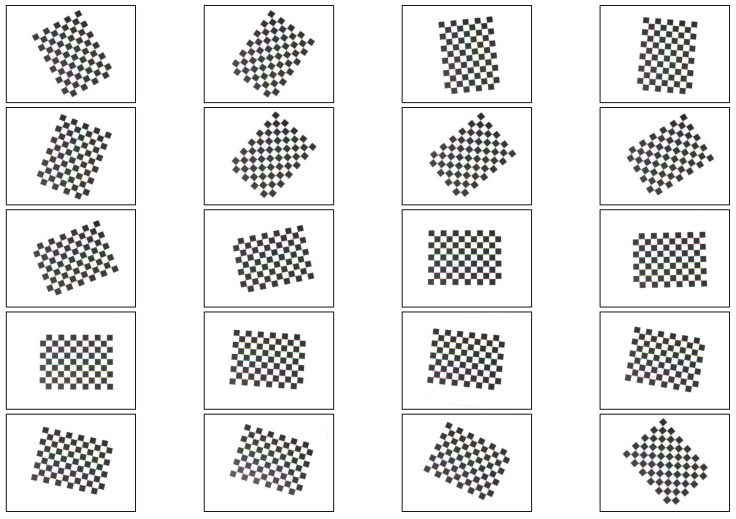
Different angle checkerboard grid calibration diagram.

**Figure 4:**
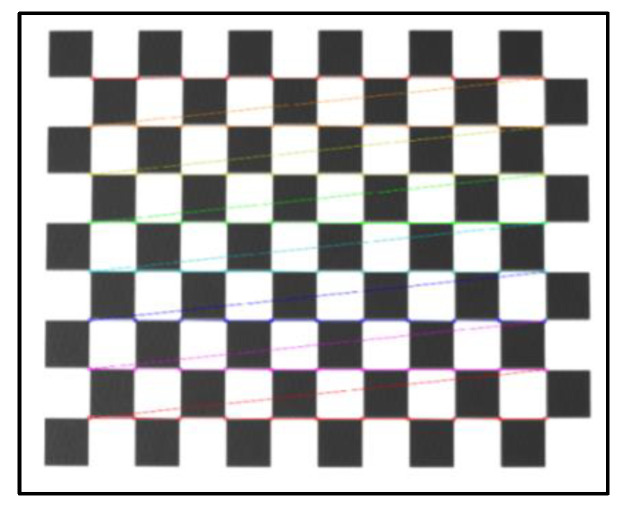
Checkerboard grid calibration diagram.

As shown in Figure 3 and Figure 4, a 12 × 9 float glass tessellation calibration template with a processing accuracy of 0.01 mm and a cell side length of 10 mm was selected. Twenty tessellated grid images were acquired according to different tilt angles. By detecting the coordinate values of sub-pixel corner points, the internal reference matrix and aberration coefficient of the camera were calculated. The original image matrix was transformed to eliminate the effects of radial and tangential aberrations. The final correction processing of the images was realized.

The internal reference matrix of the camera was derived after calibration.

(5) $$Mtx=\left[\begin{array}{ccc} f_{x} & 0 & c_{x}\\ 0 & f_{y} & c_{y}\\ 0 & 0 & 1 \end{array}\right]=\left[\begin{array}{ccc} 3273.9272 & 0.0000 & 581.4176\\ 0.0000 & 3265.1508 & 519.8155\\ 0.0000 & 0.0000 & 1.0000 \end{array}\right]$$

where $f_{x}$ and $f_{y}$ are the products of the physical focal length and cell size, and $c_{x}$ and $c_{y}$ are the pixel differences in the x and y directions between the image center coordinates and the origin coordinates.

The aberration parameter matrix was calculated to be:

(6) $$D\;=\;\left[K_{1}{,K}_{2}{,P}_{1}{,P}_{2}{,K}_{3}]\;=\;[-0.40098\;7.98644\;-0.00578\;0.00412\;-0.02106\right]$$

where $K_{1}$, $K_{2}$, and $K_{3}$ are the radial distortion parameters, $P_{1}$ and $P_{2}$ are the tangential distortion parameters.

**Figure 5:**
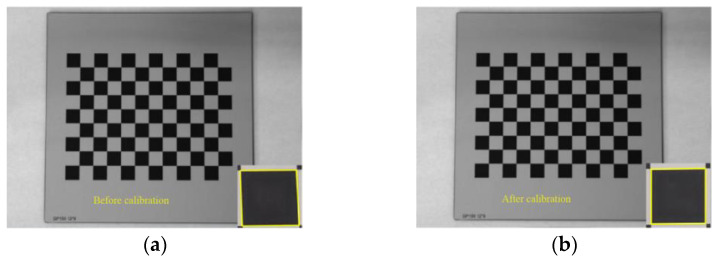
Camera calibration image: (**a**) before calibration and (**b**) after calibration.

The average reprojection error is generally considered to be less than 0.5 pixels, which indicates a high calibration accuracy. The average reprojection error of this calibration was 0.0128 pixels, which meets the requirements of camera calibration. As shown in Figure 5, the calibration plate images before and after calibration were compared. It can be seen that the corrected images obviously reduce the influence of distortion.

### 2.3. Pixel Equivalent

**Figure 6:**
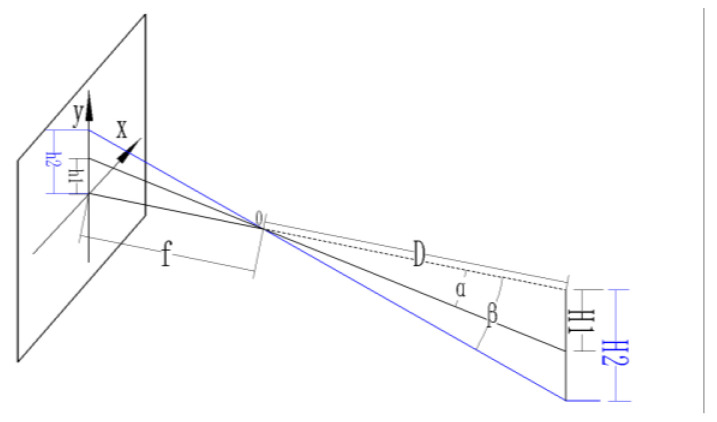
Camera imaging model.

After correcting the aberrations of the above camera, the image features could be acquired more accurately. At this point, the unit of distance detection for the object to be measured was the pixel value. In order to obtain the true size of the feature distance in the subsequently acquired image, a conversion between the pixel size and true size was, therefore, required. The imaging model of the camera is shown in Figure 6. This leads to *H2/h2 = D/f*, where *H2* is the physical length, *h2* is the pixel length, *D* is the distance between the camera and the photographed object, and f is the focal length of the camera. When the distance is fixed, the ratio of *D* to *f* is a fixed value, and the correspondence between the physical length and the pixel can be derived as a constant, which is the pixel equivalent, noted as *K*. When the pixel distance of h2 is known, the true distance of *H2* can be derived from *h2 × K*.

**Figure 7:**
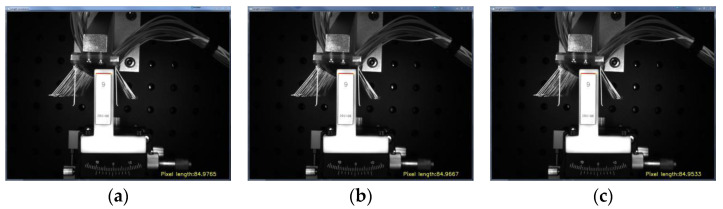
Measurement results of block length: (**a**) The 1st experiment, (**b**) the 4th experiment, and (**c**) the 8th experiment.

In this paper, a standard block measuring 9 mm × 9 mm × 30 mm was used, and the number of edge pixels of the block at the current shooting distance was obtained by image processing. The calibration value of the pixel equivalent was obtained by calculating the ratio of the real size of the standard block to the acquired pixel size. As shown in Figure 7, the figure takes the results of one, four, and eight measurements during the measurement process. The average pixel length of the block in the current state can be calculated as 84.9634 pixels from the 8 measured experimental data points, which further results in the pixel equivalent value K = 9/84.9634 = 0.1059.

To further verify the accuracy of the obtained pixel equivalent values, the upper edge of the block was measured by changing the pose of the block, and the block size was measured in reverse by the above calculated pixel equivalents. The details are shown in Table 1. Eight experiments were conducted separately. The experimental results show that the average error of the block size values obtained by pixel equivalence was 0.027, and the error value was less than 0.05 mm. The K value was relatively stable. For brush angle measurement, we adjusted the lift table to put the surface of the measuring block and the surface of the brush plate positioning block at the same horizontal position. If there was a large movement in the z-direction, it was necessary to recalibrate to obtain the pixel equivalents.

### 2.4. Image Pre-Processing

#### 2.4.1. Image Filtering

**Figure 8:**
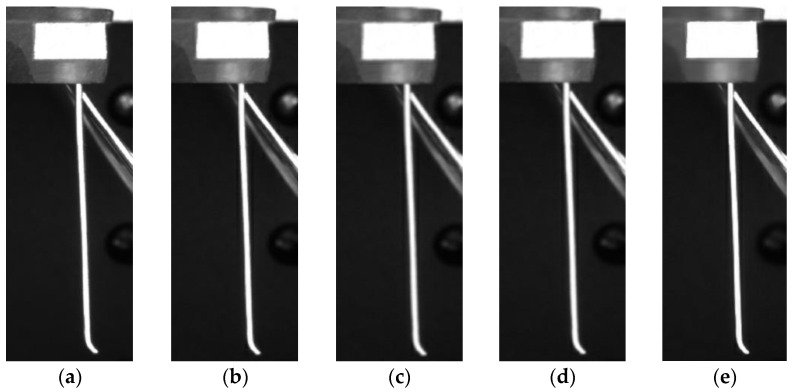
Brush filtration comparison images: (**a**) Original image and the (**b**) median, (**c**) mean, (**d**) Gaussian, and (**e**) bilateral filtering methods.

The general filtering methods median filtering, mean filtering, Gaussian filtering, and bilateral filtering were used [26]. As shown in Figure 8 below, the bilateral filtering ensured that the image edge features were preserved while the noise was suppressed. The remaining three filtering methods started to blur the edges of the brushes and were not applicable to the experimental study in this paper. In summary, the bilateral filtering method was selected for the noise reduction of the acquired bristle images.

**Figure 9:**
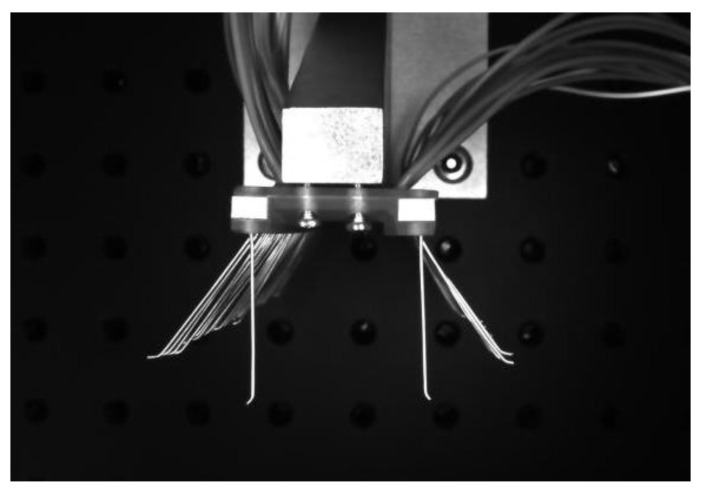
Bilateral filtering diagram.

**Figure 10:**
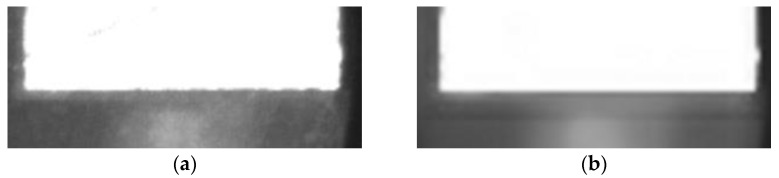
Bilateral filtering local comparison image: (**a**) Original partial picture of the brush wire, (**b**) Partial picture of the bilateral filter brush wire.

Bilateral filtering is an improved filtering method based on Gaussian filtering. This method maintains the edges of the image and removes Gaussian noise from it. Bilateral filtering is a kind of nonlinear weighted Gaussian filtering that uses the estimated value of large pixels at the edges of the image as the values of edge points. Bilateral filtering has a good edge processing effect and can retain the edge features well. As shown in Figure 9 and Figure 10 below, bilateral filtering was applied to the acquired bristle images, and it can be seen that the edge details of the bristles are obvious and the noise is effectively reduced by bilateral filtering.

#### 2.4.2. Image Edge Detection

**Figure 11:**
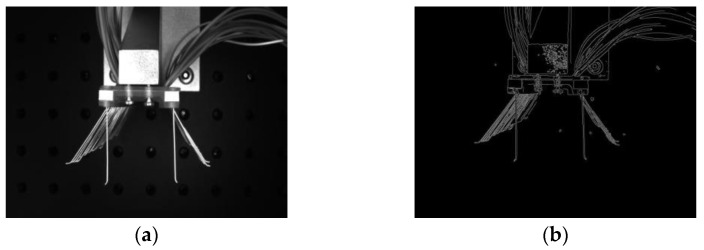
Edge detection comparison image: (**a**) Original image and (**b**) Canny edge detection.

In order to ensure accuracy in image recognition and measurement, the detection of feature edges is required for preprocessed images. In the detection process, a pixel point with a gray value greater than a threshold is an edge, and a pixel point with a value less than that threshold is not an edge [27,28]. The more commonly used edge detection algorithms are the Sobel, Laplace, and Canny algorithms. Sobel edge detection is more effective in cases where there is more noise in the image. Laplace edge detection can be used to determine the bright and dark areas of the image. Canny edge detection can effectively resist the interference of noise and detect the true image. Therefore, the Canny edge detection algorithm was chosen to detect the edges of the image in this paper. From Figure 11, it can be seen that after Canny edge detection, the edges of the brush wire and the positioning block of the brush plate were well reflected. 

#### 2.4.3. Corner Point Detection

Shi-Tomasi corner point detection is an improved algorithm based on Harris corner point detection. The principle is to select the smallest eigenvalue in the matrix and then judge this eigenvalue. If the eigenvalue is greater than a threshold, this value is judged to be a strong corner point.

The weighted autocorrelation function of the algorithm:

(7) $$F\left(x,y\right)=\underset{x_{i},y_{i}\in W}{\sum }w\left(x_{i},y_{i}\right){\left[I\left(x_{i},y_{i}\right)-I\left(x_{i}+\Delta x,y_{i}+\Delta y\right)\right]}^{2}$$

where $w\left(x_{i},y_{i}\right)$ are the weights, and the autocorrelation matrix, *A*, is given by the following equation:

(8) $$A=\left[\begin{array}{cc} \underset{w}{\sum }wI_{x}^{2} & \underset{w}{\sum }wI_{x}I_{y}\\ \underset{w}{\sum }wI_{x}I_{y} & \underset{w}{\sum }wI_{y}^{2} \end{array}\right]$$

Target validation relationship equation:

(9) $$min(\lambda_{1},\lambda_{2})>\lambda$$

where $\lambda_{1}$ and $\lambda_{2}$ are eigenvalues, and λ is a predefined threshold value, which is calculated to yield a strong corner point if this above equation is satisfied.

**Figure 12:**
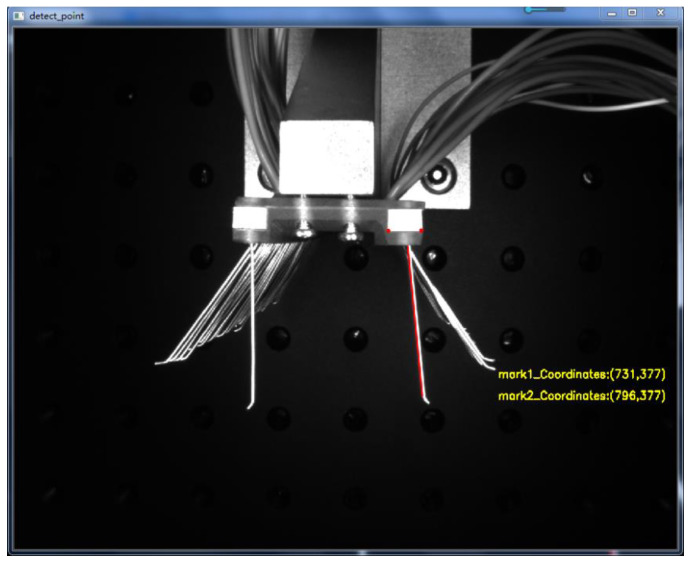
Brush wire corner point and edge straight line inspection diagram.

Compared with straight line detection in images, corner point detection has less influence under different lighting conditions and has the advantage of being almost invariant when the image is rotated at different angles. The corner points retain important local detail information during image acquisition, and the corner-point-based detection information has a certain degree of accuracy. As shown in Figure 12, the position of the corner point of the positioning block on one side of the brushboard was detected, and the detection results yielded the pixel coordinates of (pixel 731, pixel 377) for the left corner point and (pixel 796, pixel 377) for the right corner point. The edge straight line detection was also performed by constructing a mask for the brush wires to be detected. Based on the Hoff straight line detection algorithm, the left edge of the brush filament was a detected straight line, and the detection results are shown in Figure 12.

## 3. Results and Discussion

### 3.1. Principle of Bristle Angle Measurement

**Figure 13:**
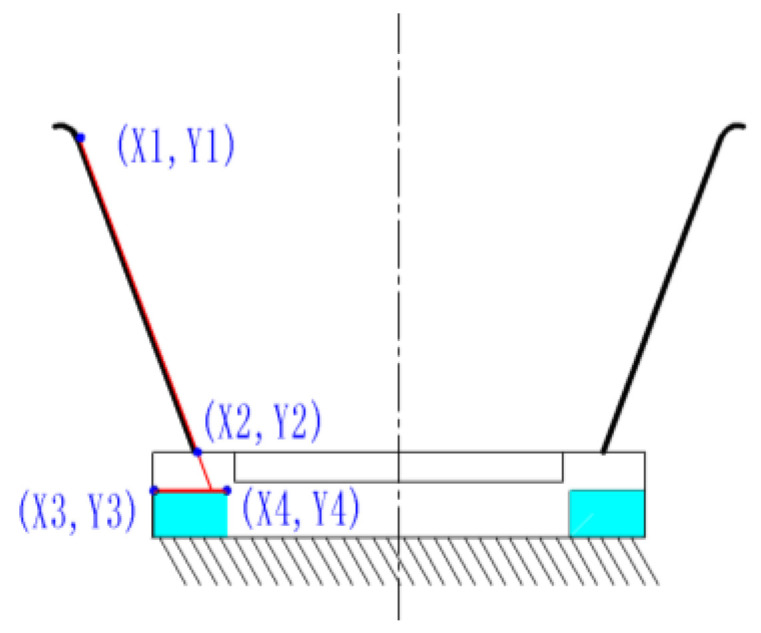
Principle diagram of brush wire angle measurement.

**Figure 14:**
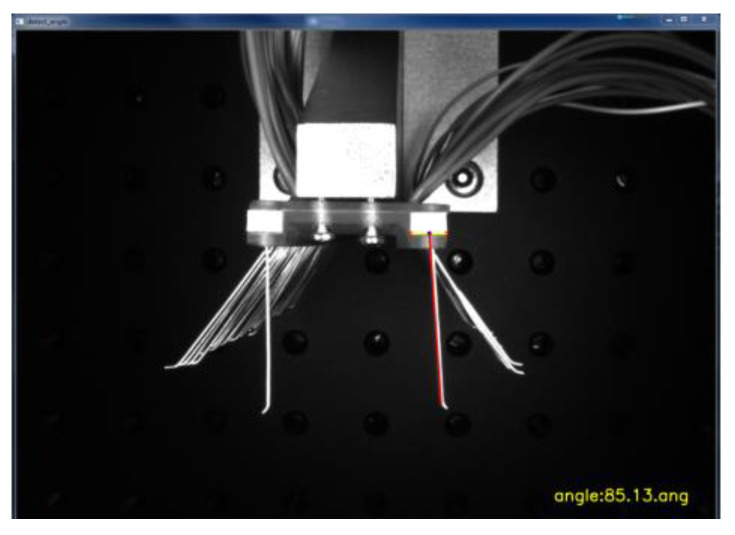
Brush wire clamping angle value detection diagram.

As shown in Figure 13, by obtaining the values of the four coordinate points in the image, the slope $k_{1}$ of the line at the detection edge of the brush wire and the slope $k_{2}$ of the line where the two corner points of the positioning block were located were calculated, respectively. The line where these two corner points were located was used as the reference line. The angle value between the two lines was further calculated by the tangent formula, and the resulting angle value was the actual angle at which the brush wire was tilted. As shown in Figure 14, the angle value of the brush wire was tested, and the angle of the brush wire was 85.13° at this time.

(10) $$k_{1}=\frac{y_{2}-y_{1}}{x_{2}-x_{1}}$$

(11) $$k_{2}=\frac{y_{4}-y_{3}}{x_{4}-x_{3}}$$

(12) $$k=tan\alpha =tan\left|\alpha_{1}-\alpha_{2}\right|=\frac{tan\alpha_{1}-tan\alpha_{2}}{1+tan\alpha_{1}tan\alpha_{2}}=\left|\frac{k_{1}-k_{2}}{1+k_{1}k_{2}}\right|$$

(13) angle=arctan(k)×57.3

### 3.2. Principle of Bristle Angle Measurement

**Figure 15:**
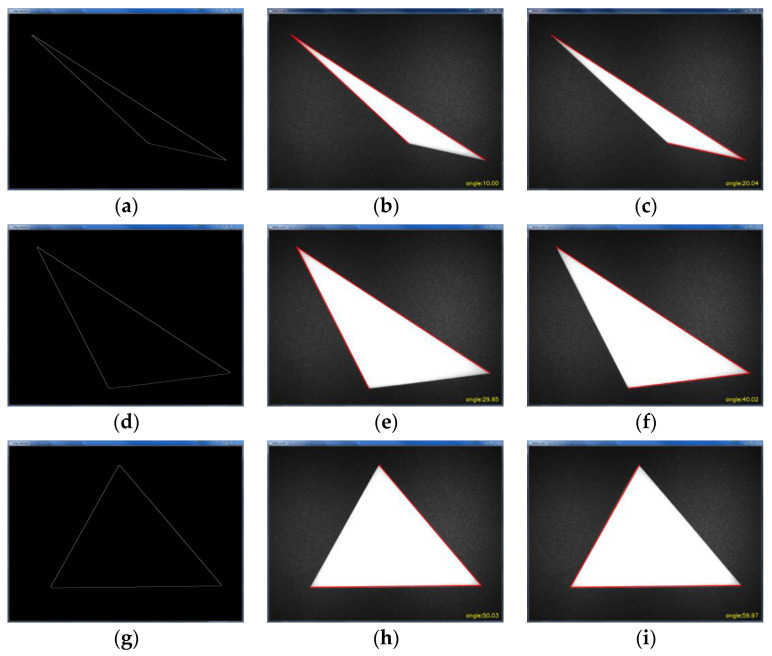
Angular value detection diagram: (**a**) First edge detection, (**b**) 10° angle detection, (**c**) 20° angle detection, (**d**) Second edge detection, (**e**) 30° angle detection, (**f**) 40° angle detection, (**g**) Third edge detection, (**h**) 50° angle detection, (**i**) 60° angle detection.

In order to test the above brush wire angle detection algorithm, three different angles of photosensitive resin triangles were made in this paper to verify the angle value detection. As shown in Figure 15, according to the calculation method of the brush wire angle. When measuring angle A, first, the linear coordinates of the adjacent side, m, of angle A were obtained by the Hoff linear detection, and then the endpoint of the other adjacent side, n, of angle A was detected to obtain the endpoint coordinate value of n. Using the neighboring edge, n, as the reference line, the angle value between the two lines m and n was obtained, and this value was the degree of angle A.

As shown in Figure 15, the outline of the triangle was first obtained using Canny edge detection. Then, the angle values were detected for three different angles of the triangle, respectively. The measurement results are shown in Table 2. The pixel coordinate values of each triangle vertex were obtained separately to determine the angle positions, and the angle detection was performed for 10°, 20°, 30°, 40°, 50°, and 60°, respectively. The data in Table 2 show that the measurement error is within 0.05°, which meets the requirements of this paper for brush wire angle detection.

### 3.3. Brush Wire Springback Control

**Figure 16:**
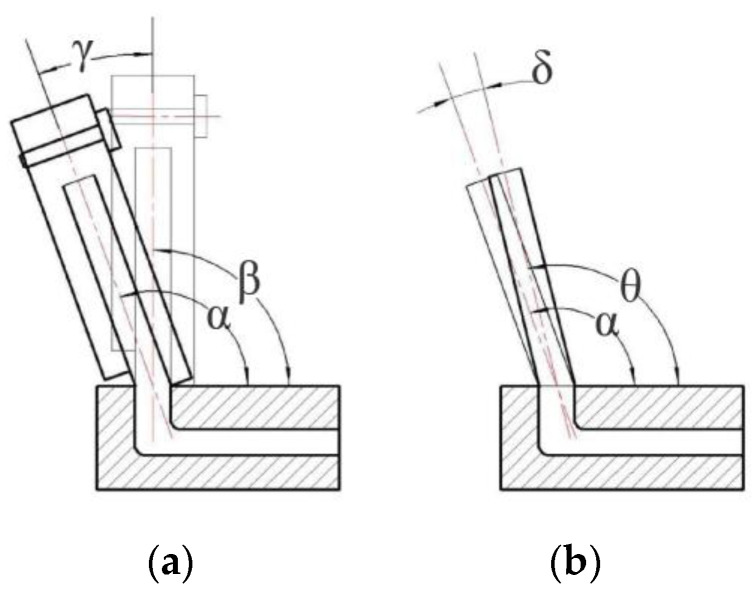
Schematic diagram of brush wire rebound: (**a**) Wire forming process, (**b**) wire rebound process.

The deformation of the brush wire during the bending process can be divided into two parts, namely, the elastic deformation stage and the plastic deformation stage. As shown in Figure 16a, the initial angle of the brush wire before forming is β, and the bending deformation is produced by the action of the deflector plate and the clamping plate. The forming angle is α and the deflection angle of the deflector plate and the clamping plate is γ. This is the angle forming stage of the brush wire. When the angle forming stage was completed, the deflection plate and the clamping plate were unloaded, and the plastic deformation of the brush wire disappeared gradually due to the high elasticity of the brush wire itself. As the elasticity was restored, the material on the pulled side of the brush wire shrank and the material on the other side elongated, resulting in the forming angle of the brush wire being smaller than the theoretical forming angle, which is called the springback phenomenon of the brush wire. As shown in Figure 16b, when the deflection plate and clamping plate were unloaded, the brush wire produced a certain amount of springback. θ is the forming angle after the brush rebound, and the springback angle is δ, which seriously affects the accuracy of brush wire forming.

#### 3.3.1. Brush Wire Rebound Angle Value Detection

**Figure 17:**
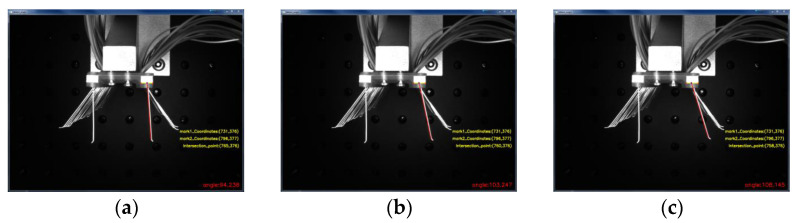
Measured angular values of bristles after rebound: (**a**) 102° springback value, (**b**) 113° springback value, (**c**) 119° springback value.

According to the algorithm of brush wire angle measurement, the angle of the brush wire after forming was measured separately. As shown in Figure 17 below, after bending the wire at a certain angle with the deflector plate, the angle of the wire was measured by unloading the deflector plate and the clamping plate. In order to compare the angle value of the rebound of the brush wire, the angle values after the rebound of the brush wire were 102°, 113°, and 119°, respectively, which were 94.238°, 103.247°, and 108.145°. It is obvious that the forming angle of the brush wire deviates greatly from the angle after the rebound, so the control of the rebound of the brush wire is very important to improve the accuracy of the forming angle of the brush wire.

#### 3.3.2. Brush Wire Rebound Angle Value Detection

The center of deflection is located at the neutral layer to build a pre-compensation model for brush wire rebound and to measure the brush wire rebound value to obtain the correspondence between the target forming angle and the overbending pre-compensation angle by experimental method. The range of wire forming was 0–30°, and the angle of wire forming was measured in 1° steps.

**Figure 18:**
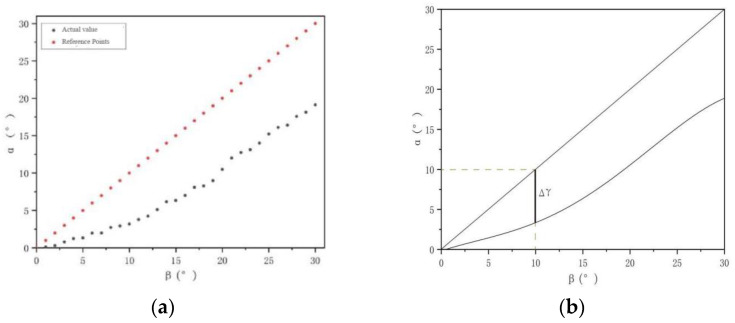
Fitting diagram of brush wire rebound: (**a**) Measured angle of brush wire rebound, (**b**) Fitted angle of rebound.

**Figure 19:**
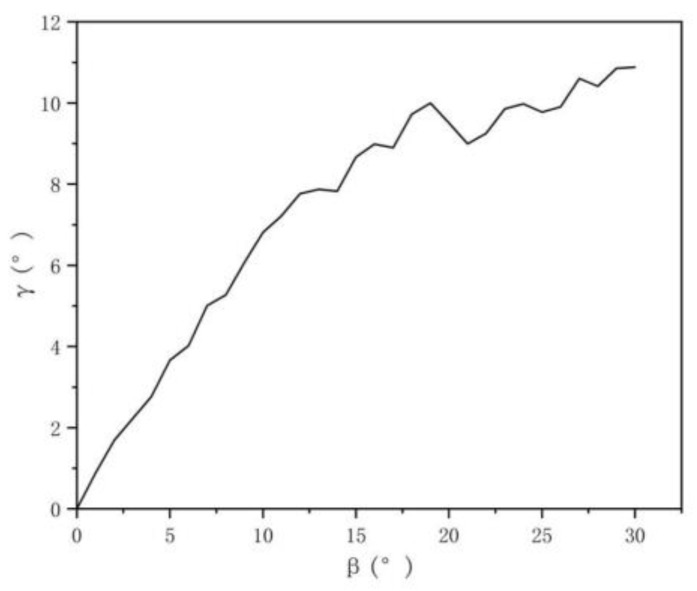
Rebound compensation value image.

α is the angle of the brush wire after rebound, and β is the forming angle of the brush wire. If the forming angle of the brush wire is 10°, the overbend of the forming angle of the brush wire is Δγ, as shown in Figure 18, and the forming angle of the brush wire should be controlled at 10° + Δγ to ensure that the forming angle of the brush wire remains around 10°. As shown in Figure 19, it is the rebound compensation value of the brush wire that is used as a reference for the calculation of the angle compensation value of the brush wire.

The function model was obtained by fitting a quadratic polynomial to the data obtained for the angle of the formed brush wire. In this model, the independent variable x is the forming angle value of brush wire, and y1 is the angle value of the forming after the spring back of the brush wire. The actual rebound y value of the brush wire was obtained by subtracting the formed angle value from the rebound angle value, i.e., the model of the function model (15) of the brush wire rebound precompensation, by which the angle precompensation value of the brush wire can be calculated at any angle from 0° to 30°. The functional model is shown as follows.

(14) $$y_{1}=-0.17159+0.38742x-0.02446x^{2}+0.0026x^{3}-0.00005036x^{4}$$

(15) $$y=x-y_{1}=0.17159+0.61258x+0.02446x^{2}-0.0026x^{3}+0.00005036x^{4}$$

### 3.4. Brushing Angle Verification

Based on the brush wire pre-compensation model derived above, the model was experimentally verified by forming the brush wire angles of 5°, 10°, 15°, 20°, 25°, and 30°. The angle forming process was performed using the above proposed overbending pre-compensation model. The experiment was repeated five times for each angle. The angle values were measured by visual measurement after the brush wire angle forming was completed. Table 3 shows the experimental data from the five repetitions of the experiment. The average value was calculated for each forming angle. It can be seen that the average value of the rebound increases with an increase in the forming angle.

In statistics, a confidence interval (CI) for a probability sample is an estimate in the form of an interval for an unknown parameter value in the parametric distribution of the aggregate that produced the sample. In contrast to point estimation, which uses a sample statistic to estimate a parameter value, a confidence interval also contains information about the precision of the estimate. The 95% confidence interval is an interval extrapolation indicator of the overall mean by the mean of the specimen data.

(16) $$P\left(\mu -1.96\frac{\sigma }{\sqrt{n}}\leq M\leq \mu +1.96\frac{\sigma }{\sqrt{n}}\right)\approx 0.95$$

where μ is the mean value of brush rebound for each group, σ is the standard deviation, and n is the number of tests.

As shown in Table 4, the rebound angle of the brush wire was calculated according to the pre-compensation model given in this paper. Combined with the mean value of the springback calculated in Table 3, the angle forming compensation model proposed in this paper was verified. The calculated value of the compensation model has a 95% confidence interval, which is more accurate in predicting the rebound angle of the bristles and reduces the angle error caused by the rebound phenomenon during the angle forming process of the bristles. However, when the angle is too large, the rebound angle is smaller than the theoretical calculated value. This is due to the plastic deformation of the bristles when the angle is too large during the angle forming process.

The method for calculating the angle of the brush filament was established by filtering and edge detection of the image. After testing, the error of the measurement result was within 0.05°. Based on this method, an experimental device of brush wire angle forming was built. Finally, a pre-compensated model of brush wire rebound was established to measure the angle of brush wire forming and rebound angle based on visual measurement. The corresponding data relationship between the angle forming value and the rebound value was obtained, the model was experimentally analyzed, and it was verified that the influence of the forming angle error caused by the rebound of the brush wire can be eliminated.

## 4. Conclusions

In this paper, the process of forming the angle of the brush wire was studied and a method of forming the angle of the brush wire was proposed. Based on this method, a brush angle forming and measuring device was built, which realizes the accurate control of the angle output value during the forming process of the brush angle. The details are as follows:

1. The camera was calibrated before the measurement experiment. The average reprojection error by calibration was 0.0128 pixels, which effectively reduced the influence of camera distortion on angle measurement. The molding angle was precisely controlled by deflecting the angular stage. In the visual measurement process, the acquired images were filtered and edge detected, and a method of calculating the brush angle was proposed. After the measurement test, the error of the measured brush angle value based on this method was within 0.05°, which meets the requirements of brush angle detection.

2. A pre-compensation model of brush filament rebound was established based on curve fitting. Based on machine vision, we measured the forming angle and rebound angle of the brush filament and obtained the corresponding relationship between the forming angle and rebound value. The correctness of the model was verified by comparing the experimental data with the real wire rebound angle. The calculated value of the compensation model had a 95% confidence interval, which was more accurate in predicting the rebound angle of the bristles. The model can be implemented reliably to optimize the brush wire forming process and improve slip ring life and contact reliability.

## Figures and Tables

**Table 1:** Block pixel size measurement results (mm).

Number	1	2	3	4	5	6	7	8	Average
Block size	9.016	9.032	8.979	9.087	9.004	8.983	9.027	9.094	9.027
Error	0.016	0.032	0.021	0.087	0.004	0.017	0.027	0.094	0.027

**Table 2:** Angle value detection results.

Number	Theoretical Value/°	Vertex X Coordinate/Pixel	Vertex Y Coordinate/Pixel	Detection Value/°	Error/°
1	10	127	119	10	0
2	20	1204	813	20.04	0.04
3	30	155	95	29.95	0.05
4	40	1230	796	40.02	0.02
5	50	1181	775	50.03	0.03
6	60	614	106	59.97	0.03

**Table 3:** Results of the brushing angle forming rebound experiments.

Angle Forming Value/°	1	2	3	4	5	Mean Value of Rebound/°
5	3.664	3.525	3.492	3.667	3.701	3.609
10	6.811	6.771	6.903	6.845	6.779	6.821
15	8.667	8.562	8.978	8.523	8.659	8.677
20	9.511	9.403	9.578	9.593	9.499	9.516
25	9.771	9.821	9.716	9.668	9.709	9.737
30	10.882	10.929	10.887	11.172	10.936	10.961

**Table 4:** Experimental results of the brush angle forming pre-compensation model.

Angle Forming Value/°	Average Value of Brush Wire Rebound	Calculated Value of the Compensation Model	95% CI
5	3.609	3.552	[3.527, 3.691]
10	6.821	6.782	[6.774, 6.868]
15	8.677	8.638	[8.520, 8.8339]
20	9.516	9.465	[9.449, 9.583]
25	9.737	9.821	[9.597, 9.877]
30	10.961	11.154	[10.936, 10.986]
